# Aggregation and Conformational Changes in Native and Thermally Treated Polyphenol Oxidase From Apple Juice (*Malus domestica*)

**DOI:** 10.3389/fchem.2018.00203

**Published:** 2018-06-05

**Authors:** Ayesha Murtaza, Zafarullah Muhammad, Aamir Iqbal, Rabia Ramzan, Yan Liu, Siyi Pan, Wanfeng Hu

**Affiliations:** Key Laboratory of Environment Correlative Dietology, Ministry of Education, College of Food Science and Technology, Huazhong Agriculture University, Wuhan, China

**Keywords:** activity center, thermal processing (TP), λmax, catechol, pyrogallol, conformation, aggregation

## Abstract

This study investigated the effects of heat treatment after purification on dissociation, aggregation, and structural modification of polyphenol oxidase (PPO) activity from apple (*Malus domestica*) juice. PPO activity at the 70°C for 10 min was still activated and drastically decreased since 20–60 min with catechol and pyrogallol as substrate. Moreover, spectral results of fluorescence and circular dichroism (CD) indicated that increasing temperature for shorter and longer durations can cause reorganization of the secondary structure of PPO and demolished the native configuration of PPO respectively. Compared with native PPO, all thermally treated PPO showed reduced activity with gradually increasing particle size shift toward section III of some fully assembled proteins treated at 70°C for 10 min (2,670 nm). Polyacrylamide gel electrophoresis (PAGE) analysis also exhibited the increase in protein content at the 70°C for 10 min with molecular size 35 kDa (7.7 ± 0.016c). Hence, thermally treated juice subjected to purification at high temperature for a short time could induce the aggregation of protein and is not really effective for PPO inactivation. For PPO, higher degree of long duration can induce the inactivation of the enzyme after processing.

## Introduction

Apple (*Malus domestica*) is commonly considered one of the most popular fruits worldwide due to its flavor, delicacy, nutritive value, and importance as a source of micronutrients and bioactive compounds, which provide beneficial health effects (Zhang et al., 2016). However, browning is the major issue for the apple fresh cut and processed products such as juices, concentrates, nectar, etc. Browning reactions are chemical processes which are triggered by the PPO, (EC 1.14.18.1) (monophenol and dihydroxy-phenylalanine/oxygen oxidoreductase) during exposure to bacterial invasions as well as mechanical injuries throughout handling and processing. Thus, technological strategies should be developed to abate enzymatic browning. PPO, a widely distributed copper-containing enzyme, in the existence of molecular oxygen catalyses two different reactions hydroxylation of monophenolase (monophenols to *o*-diphenols) and oxidation of diphenolase (*o*-diphenols to *o*-quinones), which subsequently polymerise into black, brown, or red pigments (Siddiq and Dolan, 2017). In spite of color changes, enzymatic browning also causes off-flavor and reduction of nutritional value. Therefore, these changes can be regarded as the potent hindrances for the apple processing and ultimately decreasing the marketability of this fruit.

A thermal treatment is believed to be the conventional method for preventing quality degradation of enzyme inactivation (Terefe et al., 2016). But the combined effect of temperature and time must be optimized to increase the effectiveness in inhibiting enzymatic browning with limited alterations in sensory attributes and nutritional loss (Awuah et al., 2007; Gouzi et al., 2012). PPO activity depend on the oxygen diffusion ability and the diffusion process can be controlled by the temperature regulation. At the refrigerated condition, the low temperature prevent the diffusion of oxygen and inhibit the PPO activity. Ultrasound eventually inactivate the PPO enzyme by change in the structure of PPO. Low-frequency high-intensity ultrasound caused an inactivation effect and conformational changes of purified PPO by initial dissociation and subsequent aggregation and generate the small amount of β-sheet (Liu et al., 2017).

In many studies, the activity and kinetic approaches were conducted to elaborate the inactivation process of purified or partially purified PPO enzyme by thermal processing has been reported (Sun et al., 2008; Waliszewski et al., 2009; Gouzi et al., 2012; Goyeneche et al., 2013; De Aguiar Cipriano et al., 2015; Terefe et al., 2015) but insufficiently explained the activation mechanism of PPO enzyme. PPO secondary structure was found predominately α-helix in nature, it began to change the formation of protein-protein aggregates appeared under thermal processing above 40°C (Zhou et al., 2016). However, there is no significant research available regarding the purification of juice after thermal processing with high PPO activities and its structural characterization. Therefore, this work aimed at exploration of the changes in activity and thermal behavior of PPO extracted from apple (*M. domestica*) juice. Primarily, thermally treated juice were purified to see the characteristic deformation and aggregation of purified protein with PPO activity as well as structural analyses were conducted.

## Materials and methods

### Materials and reagents

Fresh apples (*M. domestica*) were procured from a supermarket in Wuhan, China. All reagents used were of analytical grade and purchased from Sigma-Aldrich Chemical Company (St. Louis, MO, USA). Double-distilled water was used for all solutions.

### Apple juice preparation and thermal treatment

Fresh apples were washed, cleaned and cut into pieces and stored at 4°C. Apple slices were subjected to the pulper to make juice and then homogenized with ice-cold Tris–HCl (0.5 M) buffer (pH 7.0) including 10% crosslinking polyvinylpyrrolidone in a blender for 2 min at high speeds (Soysal, 2008). The homogenate was well-kept at 4°C for 12 h. The juice was filtered through a cheese cloth, and the filtrate was centrifuged at 4,000 rpm for 15 min at 4°C (Eppendorf Centrifuge 5804, Germany). The juice was kept frozen at 4°C. Thermal treatment of the apple juice experiment was carried out at selected temperatures (30, 40, 50, 60, 70, and 80°C)at exposure times (10, 20, 30, 40, 50, and 60 min) in a water bath with a thermostat. A total of 2 mL juice was pipetted into test tubes, which were heated at predetermined temperature intervals in water bath and rapidly cooled in a refrigerator to stop thermal inactivation immediately (Gouzi et al., 2012). PPO activity was induced with both catechol and pyrogallic acid as substrates.

### PPO extraction and purification

After thermal treatment, apple juice was collected and fractionated with (NH_4_)_2_SO_4_ at 25% saturation to remove impurities and 80% saturation to precipitate the protein for 1 h with PPO activity. The sample was then centrifuged (Eppendorf Centrifuge 5804 R, Germany) at 10,000 rpm for 20 min at 4°C. The precipitate was resuspended in 0.5 mol/L Tris–HCl buffer (pH 7.0). The sample was dialysed against 0.5 mol/L Tris–HCl buffer (pH 7.0) for 24 h, and the buffer was changed after 1 h during dialysis. The supernatant containing proteins was concentrated using an ultrafilter (Millipore Co., Bedford, MA, USA) and purified using the methods reported in our previous study (Cheng et al., 2014) by using DEAE Sepharose Fast Flow and Sephacryl S-200 columns (2.6 × 30 cm^2^) preequilibrated with 0.05 M Tris–HCl buffer (pH 7.0) respectively.

### Protein content

Protein content in the purified PPO was calculated using the Bradford method. Bovine serum albumin was utilized as standard protein. Coomassie Brilliant Blue G-250 was used to stain the protein solution and maximum absorption peak was observed at 595 nm wavelength (Liu et al., 2015).

### Measurement of PPO activity

According to our former investigations, the PPO activity was analyzed using a Multiskan FC (Thermo Scientific, Waltham, MA, USA). Apple extract was assayed for PPO activity by using catechol and pyrogallol as substrates according to the description of Terefe et al. (2015) with minor modifications. Such as, concisely, 0.1 mL of catechol solution (0.1 mol/L catechol in 0.05 mol/L phosphate buffer, pH 7.0) was placed in an enzyme-linked immunosorbent assay plate. Subsequently, 0.1 mL of the sample solution was added to the plate and quickly tested using the “simple kinetic method” mode at λ = 420 nm at an interval of 30 s. The slope between substrate concentration and time was calculated. Protein concentration was measured similar to the procedure described in section Protein Content. The same procedure was performed for 0.1 mol/L pyrogallol in 0.05 mol/L phosphate buffer (pH 7.0).

$$\begin{array}{l} \text{Specific activity }=\;{\text{A}}_{420}\text{ nm}/1\text{ min}/0.1\text{ mL of enzyme solution}. \end{array}$$

Relative activity of PPO was calculated using the following equation:

$$\begin{array}{l} \text{Relative activity }\text{\%}=\left(\frac{\text{Activity of treated PPO}}{\text{Activity of untreated PPO}}\right)\;\times 100. \end{array}$$

### Electrophoresis analysis

To evaluate the purification of the protein, sodium dodecyl sulfate-polyacrylamide gel electrophoresis (SDS-PAGE) was used and non-denaturing PAGE (native PAGE) was used to predict the molecular mass of purified PPO by using a Mini-Protean 4 cell system (Bio-Rad, Hercules, USA). Constant power supplies of 100 V and 10 mA iThe sample was homogenized with 2% SDS and dithiothreitol, boiled for 5 min and then loaded onto the gel along with a marker for molecular weight. The gel was stained with 50 mL of (0.1 M) Coomassie Brilliant Blue R-250.

Two native PAGE (mass-to-charge ratio protein) was performed similarly with SDS-PAGE but exclusion of heating without SDS addition (Jang and Moon, 2011). Constant power supplies of 100 V and 10 mA used torun the gel, after thatone of the gel was stained with 50 mL (0.5 M) catechol, whereas, the other one was stained with (0.1 M) Coomassie brilliant blue R-250.

### Native and thermally treated structural measurements

#### Circular dichroism (CD) spectral measurement

CD analysis was performed according to our former literature (Huang et al., 2015; Liu et al., 2017) by using a JASCO chiroptical spectrometer (Japan Spectroscopic Co., Tokyo, Japan). Spectral analysis was performed using a 0.009 cm optical-path-length quartz at ambient temperature (25 ± 1°C) to record CD measurement. Sample of protein concentration (0.2 mg/mL) was obtained using 50 mmol/L Tris–HCl buffer (pH 7.0), blank proceeded for CD measurements. CD parameters were examined in the far ultraviolet range of 190–240 nm at 100 nm/min, 2 s of time constant and 1 nm bandwidth. The measurements were changed into mg (molar extinction coefficient difference), and CD measurement analysis was examined. Protein Secondary Structure Estimation software of Spectra Manager (Spectra Manager Version 2, JASCO, Japan) was used to estimate secondary structure of protein based on reference (Yang's equation).

#### Fluorescence spectral measurement

Fluorescence emission spectral measurements were performed using an F-4600 fluorescence spectrophotometer (Hitachi, Tokyo, Japan). Protein sample solution (0.2 mg/mL) at an emission wavelength of 350 nm was measured to obtain maximum excitation wavelength. The emission spectra of protein solutions were obtained at a wavelength range of 300–400 nm with maximal excitation wavelength (λ_ex_ = 280 nm) of protein. Both slits (Em and Ex) were measured at 10 nm (Liu et al., 2009, 2015). Scan speed was 200 nm per min; response time was 0.1 s with a step length of 1 nm for capturing data (Liu et al., 2013).

#### Particle size distribution (PSD) measurement

PSD measurements were achieved with a Zetasizer Nano ZS device (Malvern Instruments, Malvern, Worcestershire, UK) according to the description of Cheng et al. (2014) with few modifications. Such as native and thermal treated protein solutions of 0.3 mg/mL were prepared at 25°C in 50 mM phosphate buffers (pH 7.0) instead of 50 mM Tris–HCl buffers (pH 6.8) and measured for the protein PSD. A wavelength laser of 532 nm of scattering light with reflection angle of 15° at 25°C was applied to the sample solution. Final results of PSD were stated as average of five replicates.

### Statistical analysis

Data for each treatment were collected in triplicate, and their results were expressed as average ± standard deviation and analyzed using the descriptive statistics function in the Origin 8.5 software (Origin Lab, Northampton, USA).

## Results and discussion

### Thermostability of apple PPO

**Figure 1:**
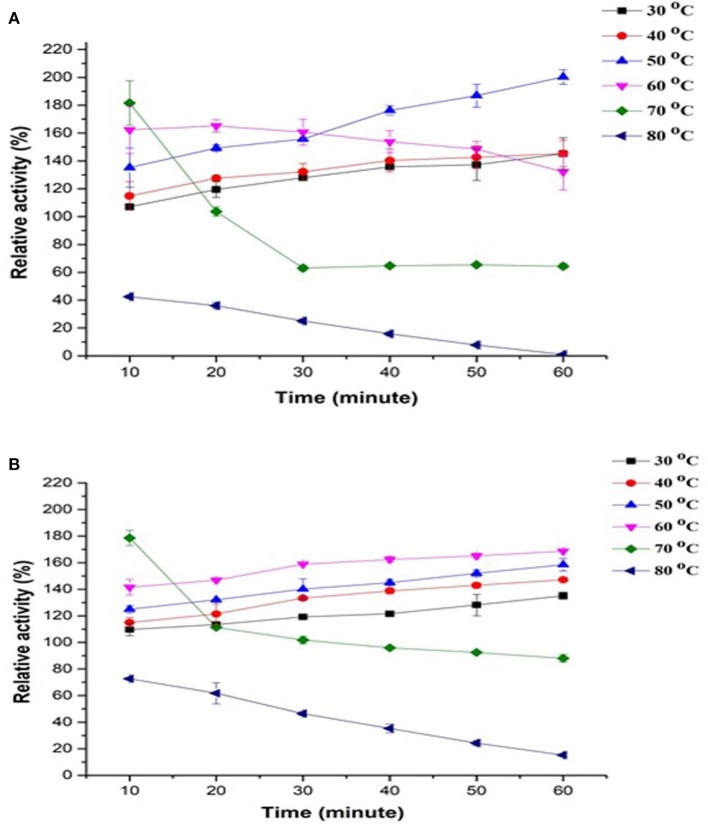
Effect of temperature stability on relative activity of crude apple juice polyphenol oxidase (PPO) by incubating at 30–80°C for 10–60 min and by using different substrates, namely, **(A)** catechol and **(B)** pyrogallol. All data were the means ± *SD*.

Thermostability of PPO in apple juice was investigated using two substrates, such as catechol (Figure 1A) and pyrogallol, (Figure 1B) at 10°C/10 min incubation at a temperature range of 30–80°C and up to 60 min. Results showed that PPO retained most of the relative activities at up to 50°C continuously for 60 min with catechol as substrate. Compared with catechol measurement, PPO remained stable at 60°C when using pyrogallol and prolonged duration. A substantial increase of up to 181–178% was observed in PPO activity with catechol at 60–70°C for 10 min, respectively. Meanwhile, the activity of PPO treated at 70°C for 20–60 min with both substrates drastically decreased. At 70°C, the highest relative activity was observed, followed by a decrease of 64–88% with catechol and pyrogallol, respectively. Inactivation of the enzyme at 60–70°C occurred, counteracting activity increase and thereby decreasing activity peak during prolonged incubation. The lowest relative activity was noted at 80°C for 10–60 min in catechol (42–0.95%) and pyrogallol (72–15%). Thus, almost complete enzyme inactivation was observed at 80°C even in a (10 min) short period (Figures 1A,B). The results indicated that optimum temperatures for PPO enzyme were 50°C with catechol and 60°C with pyrogallol under prolonged duration of incubation. Substrate structure causes a reaction between the enzyme's active site and substrate, leading to a different type and degree of inhibition of PPO activity (Goyeneche et al., 2013).

These trends explain the phenomena of simultaneous activation (because of partial conformational changes induced by heat in latent enzyme) and inactivation (due to the denaturation induced by heat) of PPO enzyme during heat treatment, with activation occurring at low temperatures and transpiring inactivation at the high temperature of 80°C. The variety influences PPO activity and properties, source and physicochemical conditions, such as temperature, pH, and climate where PPO was exposed (Aboubakar et al., 2012). In the same way, (Terefe et al., 2015) observed that latent PPO remained activated at up to 80°C for 30 min, as observed in the blueberry extract. Similarly, Soysal (2008) also reported apple (*Gloster cultivar*) PPO inactivation at 75°C and prolonged exposure time of up to 70 min. The logical reason behind PPO activation under thermal processing may be because of protein dissociation, the release of other enzymes and interactions among constituents in the extractor. Activation of latent PPO is also a reason which may have contributed to the observed effect. Some literature on the existence of latent PPO enzyme in apples are also available; this type of PPO may show an active transient form appearing during the transition from the native to inactive form of the compound (Vámos-Vigyázó and Haard, 1981) Some reports also noted an increase in activity of plant origin PPOs following thermal processing (Buckow et al., 2009), and this event was associated with activation phenomena of PPO latent precursors in PPO extracts. PPO in plant tissues is relatively heat labile and requires high temperature for inactivation. The precursor of latent PPOs has been studied in various fruits and vegetables such as, beetroot (Gandía-Herrero et al., 2005), iceberg lettuce, apples, persimmons, grapes (Chazarra et al., 2001; Núñez-Delicado et al., 2003, 2007), and peaches (Winters et al., 2008). Terefe et al. (2015) described that to activate latent precursors from the active site, extension shield required to be either removed or cleaved through limited conformational change of temperature, proteases, pH, and other physicochemical parameters.

### Thermally induced dissociation and aggregation of PPO

**Figure 2:**
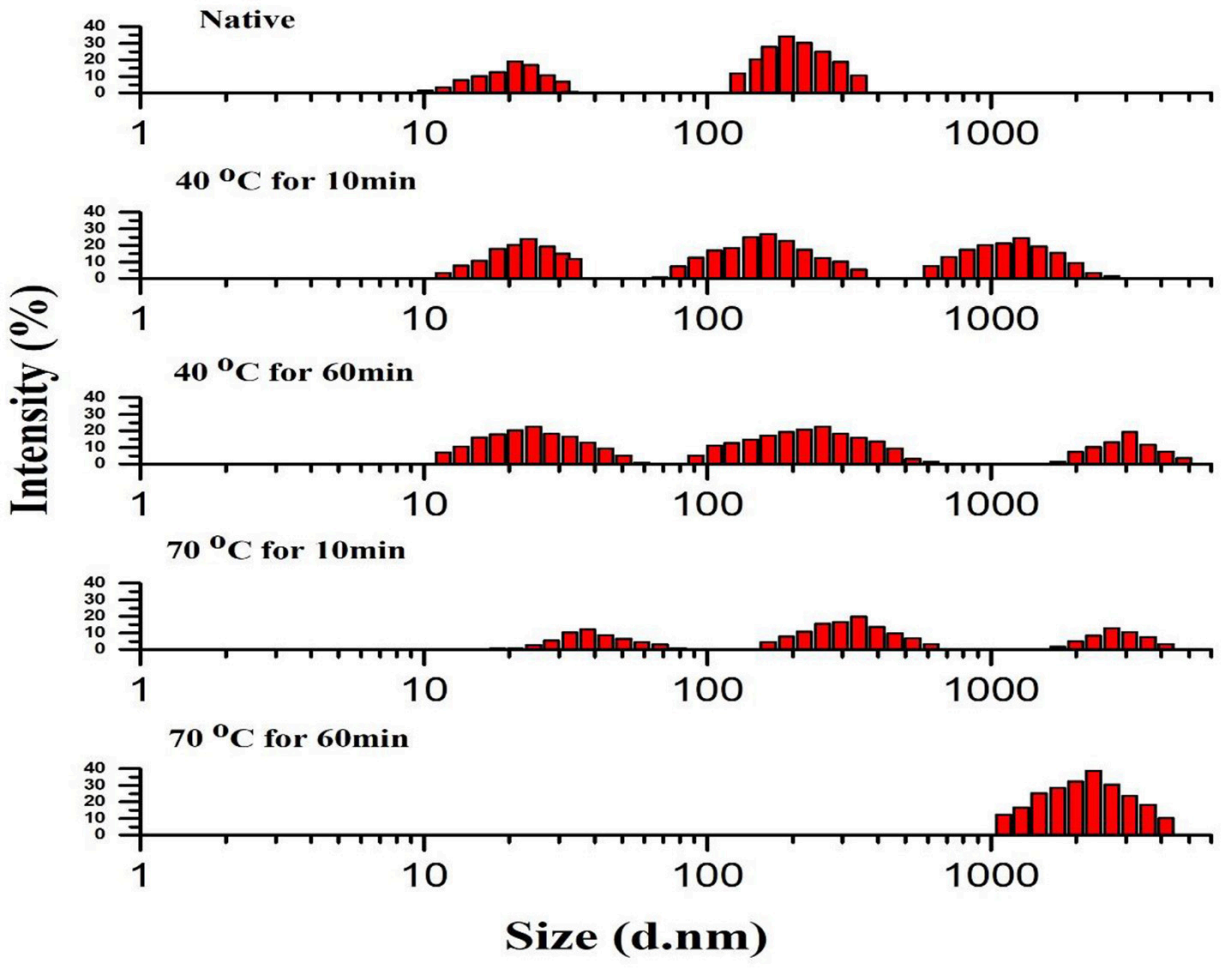
Particle size distribution (PSD) pattern of native and thermally treated PPO. All data were the means ± *SD*.

Figure 2 compares PSD patterns of purified apple PPO obtained by using thermal treatment with that of native ones. Heat treatment at two different temperatures with short exposure (10 min at 40–70°C) and prolonged exposure times (60 min at 40–70°C) displayed an extensive effect on PSD pattern of PPO.

Thermally induced dissociation and aggregation of purified PPO, as determined by PSD analysis, revealed that native purified PPO showed a relatively wide span with two sections: section I exhibited the native purified protein (21 nm) value with fraction containing 18.87% and section II of some partially aggregated protein (190 nm) with 34.06% value in number fraction. The section II possibly comprises a small quantity of PPO aggregates. Meanwhile, treatment at 70°C for 10 min showed the least intensity for all three sections as follows: section I of purified protein with thermally treated PPO treatment (37.8 nm) with number fraction occupying 12.19 %; section II of some partially gathered proteins (342 nm) with 19.72% value in number fraction; and section III of some fully assembled proteins (1720 nm) with 12.80% value in number fraction. PSD variation was obtained using PPO activity, tendencies under different thermal treatments. Compared with native PPO, thermally processed PPO showed lower activity with steadily increasing particle size.

Changes, such as fragmentation, deformation, and aggregation of PPO molecules and modification of secondary structure, affected active catalytic sites and resulted in PPO activation and inactivation. The deformed PPO molecules further aggregated, and a massive change in PPO conformation occurred at high temperature for prolonged exposure similar to those observed during thermal treatment at 70°C for 60 min. PPO was strongly inactivated and denatured at the same temperature. A recent study revealed that intermediates have slightly different in their size from the native structure of protein, but significantly showed different properties (Leeb et al., 2015).

Purified PPO protein forms aggregates (Bondos and Bicknell, 2003). Therefore, the small-size PPO may association to form protein aggregates; this phenomenon further aggregates to generate significant accumulations, thereby increasing the distribution span (Liu et al., 2017). Thermal treatment may also promote interactions among protein molecules, thereby inducing molecules to aggregate and form large particles. Furthermore, aggregated proteins may feature inner structures that are hindered from interacting with substrates for catalytic reaction. After thermal treatment, variations in particle diameters of PPO showed aggregation and dissociation. High temperatures for short and long durations under thermal treatment induced aggregation of small aggregates and dissociation of large aggregates (Li et al., 2014). Breakage of intermolecular and intramolecular polypeptide bonds may enhanced the formation of fragments from aggregates and clusters (Chandrapala et al., 2011). Thermal treatment may also have enhance the protein interactions among molecules by the availability of their catalytic side for short exposure and increase activation rate by reducing particle size, dissociation, and fragmentation of molecules.

### Analysis of conformational change in the secondary structure of PPO through thermal process by circular dichorism (CD) spectroscopy

**Figure 3:**
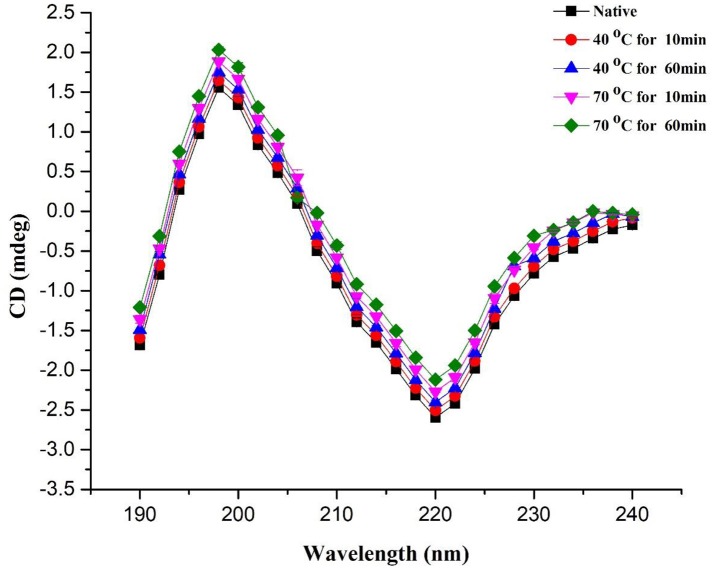
CD spectra of PPO during heating from 40 to 70°C for a short (10 min) and long periods (60 min). All data were the means ± *SD*.

Secondary structure of protein, including α-helix, β-turn, and β-sheet of PPO, were investigated by CD spectroscopy. It is a useful technique for observing peptides and polypeptide structures of proteins (Zhou et al., 2016). In this study, this technique was utilized to explore the variations in the secondary structure of PPO exposed to thermal processing. CD spectra of the native PPO displayed a negative peak (troughs) at 222 nm and a positive one at 198 nm. This discovery signified that native PPO showed a characteristic α-helix alignment in secondary constellation. CD analysis and secondary structure contents of the native and thermally purified PPO at 40–70°C for 10–60 min are shown in Figure 3 and Table 1, respectively. The results predicted that α-helix and β-turn contents decreased, whereas, β-sheet and random-coil contents increased when increasing temperature and time. Loss of α-helix by thermal treatment disorganized the structure and ultimately increased the negative ellipticity of CD spectra.

**Table 1:** Secondary structure contents of native and thermally treated PPO.

**Thermal treatment**	**Secondary structure contents**					
	**α-Helix (%)**	**β-Sheet (%)**	**β-Turn (%)**	**Random coil (%)**	**PPO activity (%)**	**Concentration (%)**
Native	37.70 ± 0.58a	28.50 ± 1.05d	17.50 ± 0.51a	16.30 ± 1.49d	18.42 ± 0.60cd	1.70 ± 0.003b
40°C for 10 min	33.70 ± 1.15ab	34.00 ± 1.52c	8.70 ± 0.92b	23.60 ± 0.66c	22.37 ± 0.74c	3.40 ± 0.005a
40°C for 60 min	31.90 ± 0.49bc	36.80 ± 0.80b	5.60 ± 1.05c	25.70 ± 1.06bc	40.59 ± 0.20b	5.00 ± 0.002bc
70°C for 10 min	32.50 ± 1.06b	38.20 ± 0.89ab	2.00 ± 1.04d	27.30 ± 0.47b	61.45 ± 0.11a	7.70 ± 0.016c
70°C for 60 min	24.4 ± 1.46c	40.6 ± 0.66a	1.60 ± 0.68e	33.40 ± 01.24a	12.72 ± 0.09d	0.50 ± 0.016d

In columns, the different letters represent the significantly difference (P < 0.05) among the values (means ± SD values)

Native PPO comprised 37.70 ± 0.58% α-helix, 28.50 ± 1.05% β-sheet, 17.50 ± 0.51% β-turn, and 16.30 ± 1.49% random coil. Increase in temperature in the random coil and β-sheet contents increased with decreasing α-helix content of PPO. When temperature was increased to 70°C for 10 min, α-helix content decreased to 32.50 ± 1.06%, whereas, β-sheet and random coil contents increased to 38.20 ± 0.89 and 27.30 ± 0.47%, respectively, compared with those obtained at 40°C for 10 min (33.70 ± 1.15% α-helix, 34.00 ± 1.52% β-sheet, 8.70 ± 0.92% β-turn, and 23.60 ± 0.66% random coil). Time also increased the negative ellipticity of CD spectra by increasing disorders in protein structure. The positive peak value at 198 nm increased to some extent with increasing treatment time. As shown in Table 1, high temperature for a short time (70°C for 10 min) showed a low loss in α-helix in CD compared with the high temperature for a long period (70°C for 60 min). A similar trend was also observed in relative activity of PPO, as illustrated in Table 1. According to results of CD analysis and PPO activity, advent of β-sheet, and loss of α-helix conformation may be a turning for preponderant-active conformation of PPO, signifying the denaturation of catalytic center. Spectral results of fluorescence and CD indicated that increasing temperature for short- and long-duration treatments can cause structural modifications of PPO. Thus, thermal processing demolished the native configuration of PPO as a result of the reorganization of secondary structure.

Ioniţǎ et al. (2014) stated that PPO is susceptible to temperature fluctuations and numerous structural changes at high temperatures. Zhou et al. (2017) reported that crude PPO contains α-helix (38.3%), β-sheet (12.7%), β-turn (25.1%), and random coils (23.9%). α-Helix content of PPO reduced with high temperature. At temperature of up to 80°C, α-helix content decreased to 20.6%, whereas, random coil and β-sheet materials rose to 36.0 and 24.3%, respectively.

Numerous reports agreed that thermal processing caused redisposition of secondary structure of PPO enzymes (Lopes et al., 2015) discovered that heat processing alters secondary configuration with a partial decrease of α-helix content of horseradish peroxidase. The α-helix contents 35.3 and 35.7% of PPO by CD analysis, respectively (Yi et al., 2012; Liu et al., 2013). Similarly, (Baltacioglu et al., 2015) described that native PPO of mushroom features α-helix of 38.87%, β-sheet of 27.91%, β-turn of 14.93%, random coil of 15.01%, and aggregated β-sheet of 3.27% using Fourier-transform infrared spectroscopy analysis. Correlating to reported data, α-helix content of PPO (38.3%) was parallel to that of previous findings.

Earlier studies reported that primarily, PPO structure comprises α-helical conformation with the active part buried in four α-helix bundles. The catalytic binuclear copper center of PPO molecule is housed in four central helix bundles, and each of the two active copper sites are lined by three histidine residues provided by the four α-helices. As a result, reshuffling of secondary structure and interruption of α-helix structure of PPO force dislocation of the active site and PPO inactivation (Decker et al., 2006; Huang et al., 2015).

### Fluorescence spectroscopy analysis for conformational tertiary structure changes of PPO

**Figure 4:**
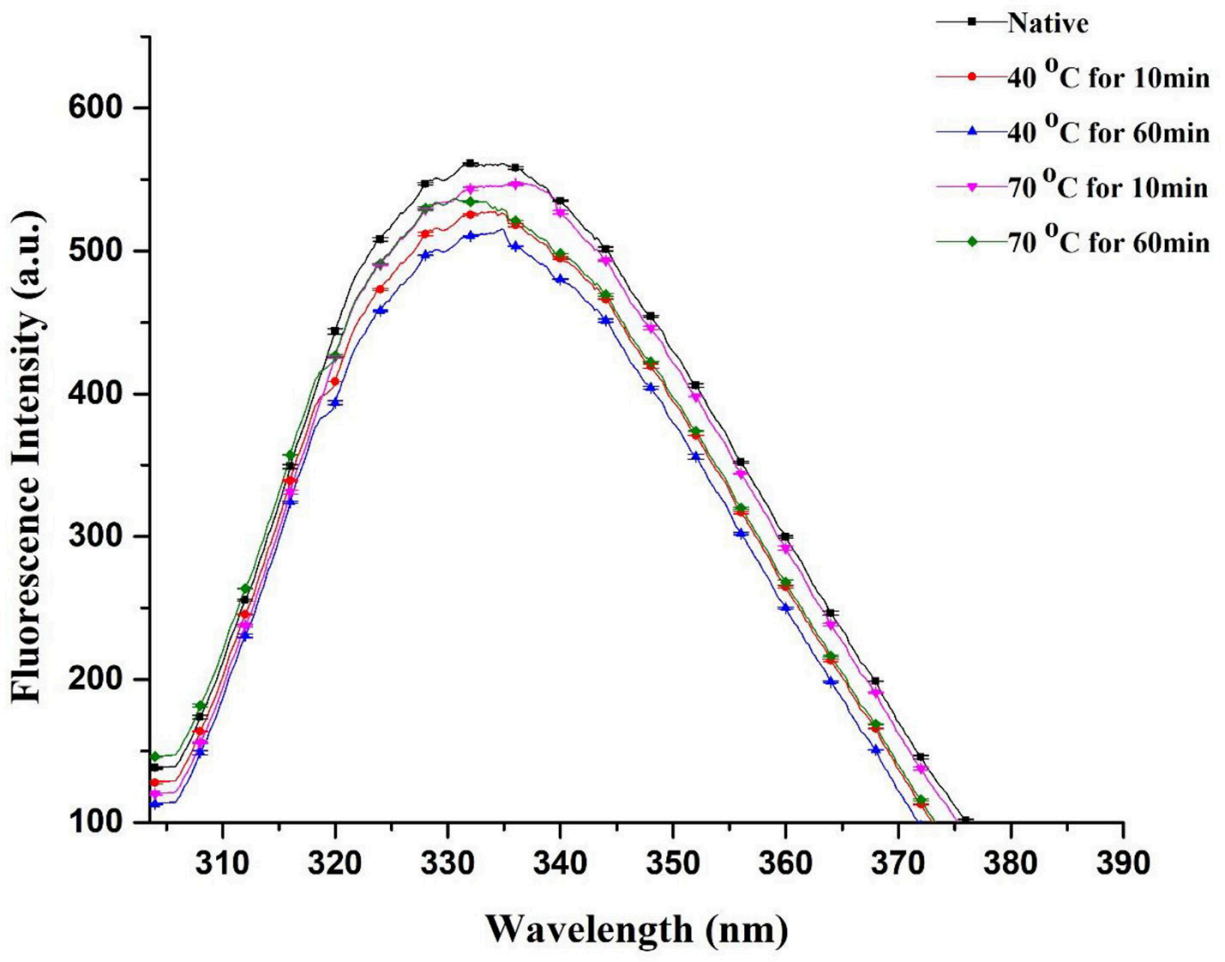
Fluorescence spectra of native and thermally treated PPO at different holding times and various temperatures and correlation analysis between redshift and inactivation rate. All data were the means ± *SD*.

Fluorescence spectral analysis is a worthwhile practice to study alterations in tertiary structures of proteins. Thus, modifications in tertiary configurations of PPO may be predicted by intrinsic fluorescence spectroscopy. However, intrinsic fluorescence of cyclic amino acid remnants is sensitive to polar environments (Viseu and Carvalho, 2004; Zhou et al., 2009). In this study, fluorescence individualities of native and thermally treated PPO were inspected using fluorescence spectroscopy. Figure 4 displays fluorescence intrinsic spectra of purified apple PPO under thermal treatment. The extreme fluorescence emission wavelength of PPO redshifted under heat processing. λmax of native PPO reached 332 nm with a fluorescence intensity of 561.4 ± 0.37. This result suggested that tryptophan remnants in PPO were situated in the nonpolar hydrophobic environment. Meanwhile, the effect of thermal treatment (40–70°C) and different time intervals (10–60 min) on λmax of purified PPO was detected. PPO fluorescence emission wavelength ranging from 334.2 to 337 nm, slightly redshifted in peak wavelengths with minute decline in fluorescence intensity during thermal processing. This finding indicates that PPO slightly unfolded, causing interruption of tertiary structure and increase in amino acid residues. Liu et al. (2017) stated that PPO was excited at 280 nm and showed that the maximal fluorescence emission wavelength of PPO showed a redshift variation as temperature raised up to 55°C. However, when the temperature was raised to 70°C for an extended treatment duration, for instance, 60 min, fluorescence spectra showed enormous changes with blue shift at 330.4 nm and PPO inactivation. During thermal treatment at 40 and 70°C, fluorescence intensity decreased than native. However, at high temperature of 70°C, fluorescence intensity (535.8 ± 0.08–547.0 ± 0.76) did not decrease similar to those at 40°C (514.8 ± 0.03–527.8 ± 0.49) and from native PPO (561.4 ± 0.37). Changes in concentration may be due to complex structural changes induced by thermal treatment when fluorophores are buried inward or exposed outward of the enzyme. As mentioned previously, λmax of native PPO decreased under thermal treatment, indicating that fluorophores moved to the inner side of the purified enzyme and subsequently caused fluorescence intensity to decrease. Thus, decreased intensity can be ascribed to bury fluorophores.

Li et al. (2007) proposed that uncovered hydrophobic groups can re-associate or conglomerate to form an extra stable structure. In a recent study, Zhou et al. (2017) discussed that at high temperatures (70–80°C), PPO may form conglomeration and avert a reduction of fluorescence intensity. Numerous aggregates replace maximal of the small groups of molecules. PPO was denatured and presented a tendency to form protein aggregates, resulting in strong effect of PPO inactivation for long thermal period. Recent correlated reports agreed with our findings and indicated that PPO was detected from one of the enzyme molecular groups, which lost their shape and formed aggregates at high temperature of 75°C. Ultimately, this phenomenon dislocated the natural PPO structure and induced unfolding or deformation of the compound (Ioniţǎ et al., 2014; Liu et al., 2017). Therefore, a sequence of fractional blue shifts was noticed with increasing temperature, and the fall in PPO fluorescent intensity induced by thermal processing was prominent; this result was in agreement with fluorescence spectrum changes of thermally processed PPO induced by disruption of its tertiary structure (Zhou et al., 2016). Hydrophobic interaction of a few unfolding particles may overcome the electrostatic obstacle for protein aggregation and consequently form aggregates (Delahaije et al., 2015). Compared with untreated PPO (18.42 ± 0.60), the lowest relative activity of 12.72 ± 0.09 was observed with thermally treated purified PPO (Table 1). This result proves enzyme inactivation by heating, causing a blue shift in intrinsic fluorescence spectra. The fluorescence spectral changes were closely related to PPO activity and PSD of molecules (Figure 4).

### PAGE analysis of PPO and its molecular weight

**Figure 5:**
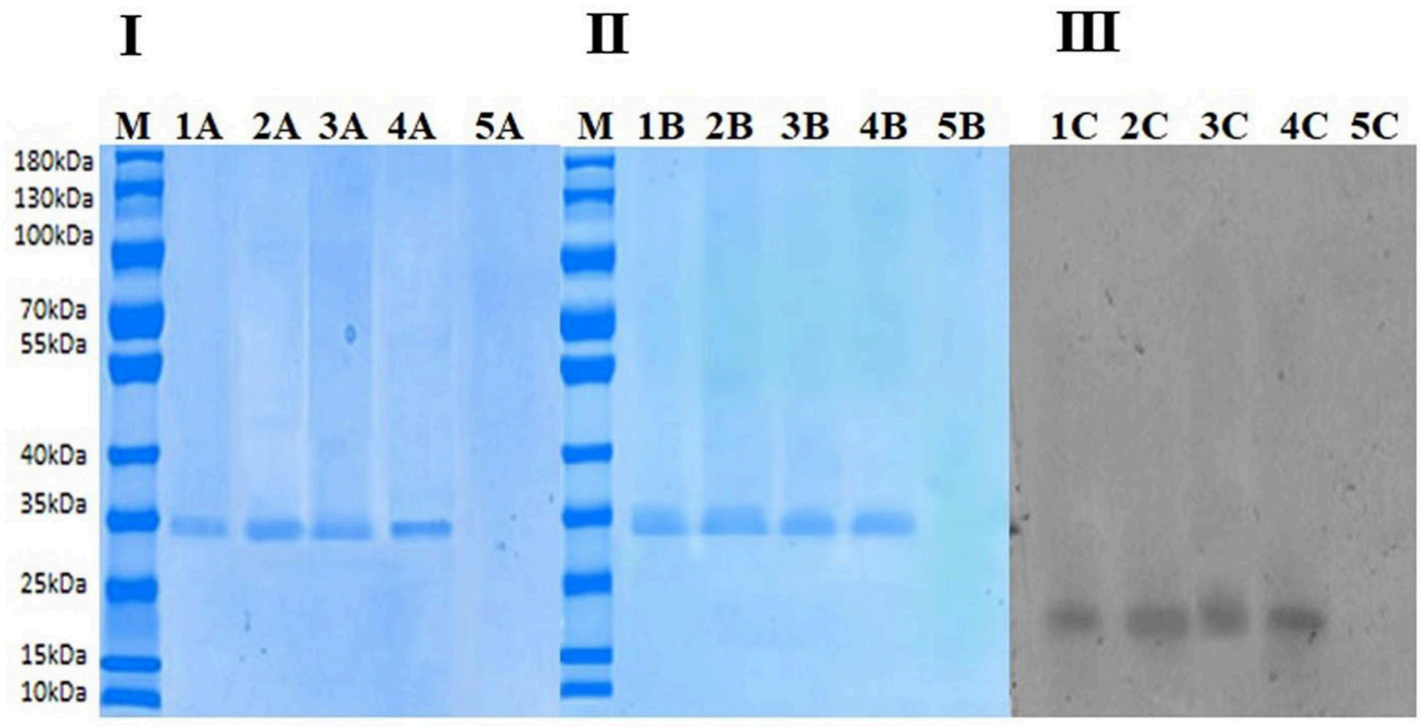
**(I)** Sodium dodecyl sulfate-polyacrylamide gel electrophoresis (SDS-PAGE). In the above figure, M indicates marker, Lane 1A indicates nonthermally treated PPO. Lane 2A shows thermally treated PPO at 40°C for 10 min. Lane 3A shows thermally treated PPO at 40°C for 60 min. Lane 4A shows thermally treated PPO at 70°C for 10 min. Lane 5A shows thermally treated PPO at 70°C for 60 min. **(II)** Native PAGE stained with Coomassie Brilliant Blue R-250. In the above figure, M indicates marker, Lane 1B indicates nonthermally treated PPO. Lane 2B shows thermally treated PPO at 40°C for 10 min. Lane 3B shows thermally treated PPO at 40°C for 60 min. Lane 4Bshows thermally treated PPO at 70°C for 10 min. Lane 5B shows thermally treated PPO at 70°C for 60 min. **(III)** Native PAGE stained with catechol. In the above figure, Lane 1C indicates non-thermally treated PPO. Lane 2C shows thermally treated PPO at 40°C for 10 min. Lane 3C shows thermally treated PPO at 40°C for 60 min. Lane 4C shows thermally treated PPO at 70°C for 10 min. Lane 5C shows thermally treated PPO at 70°C for 60 min.

Figure 5I shows SDS-PAGE of final extracted proteins treated at 40°C (10 and 60 min) and 70°C (10 and 60 min). Except at 70°C (60 min) in lane 5A, only one band for all treatments can be observed when the gel was stained with Coomassie Brilliant Blue R-250. Purity of PPO protein after final purification was analyzed by native PAGE, as shown in Figure 5II. The molecular weight of the band was estimated to be about 35 kDa, indicating protein homogeneity. PPO isoforms were observed in native and thermal treatments when the gel was stained with Coomassie Brilliant Blue R-250, indicating that these isoforms may feature similar structure except at 70°C (60 min) no band for it due to complete denaturation of enzyme. Meanwhile, when catechol was used to stain the gel for activity assay, intense activity was observed at the same protein band, confirming that the purified protein (7.70 ± 0.016 in Table 1) was similar to that with PPO activity (Figure 5III lane1C-4C). Although no enzymatic activity band of the protein was observed in lane 5B native PAGE (Figure 5II) without the addition of denaturing SDS stained with catechol; Figure 5III treatment at 70°C for 60 min), complete loss of protein was observed due to thermal degradation. Increasing temperature during thermal processing caused an increase in protein content (Table 1) at the short treatment time, as shown in Figure 5I. Lane 4A represents the highest protein concentration (7.70 ± 0.016) at 70°C for 10 min. Although enzyme inactivation occurred at high temperature for an extended time due to protein denaturation, no band was observed at 70°C (60 min), as shown in Figures 5I–III. As shown in Figure 5III, the trend for strongest activity was 40°C (10 min) < 40°C (60 min) < 70°C (10 min), as observed in native PAGE during treatment with catechol as substrate.

## Conclusion

In this study, PPO in apple juice retained most of its relative activities at up to 50°C with catechol as substrate and remained stable at 60°C when using pyrogallol as substrate. The PPO activity of thermally processed purified juice was initially activated (because of partial conformational changes induced by heat in latent enzyme) and subsequently inactivated (due to denaturation induced by heat) of PPO enzyme by increasing thermal treatment. Thermally treated purified juice at high temperatures for short and long durations induced formation of smaller aggregates and dissociation of larger aggregates. α-Helix and β-turn contents decreased, whereas, β-sheet and random coil contents increased with increasing temperature and time. Results of fluorescence spectra showed a slight red shift in peak wavelengths, indicating the disruption of tertiary structure. Thermally processed PPO molecules were stable and activated at a mild temperature for long duration, whereas high temperature caused collapse of the catalytic center, and PPO inactivation occurred due to protein aggregation. Fluorescence spectral and CD results indicated that increasing temperature for short and long duration treatments can cause structural PPO modifications. Thermal treatment demolished the native configuration of PPO as a result of the reorganization of secondary structure. This study elucidates PPO thermodenaturation and relates structural changes to PPO activity. Furthermore, this work provides a theoretical guide for further understanding the enzymatic caused browning mechanism by PPO under thermal treatment. Future works will be focused on mechanisms behind PPO activation in fruit and vegetable enzymes.

## Author contributions

The present article is the part of AM's Ph.D. thesis work. She carried out all the experiments of this study. ZM helped in writing and analyzing the results. RR supported technically and gave big contribution in statistical analyses. AI helped in writing the manuscript. YL gave a helping hand while the experiments were being carried out. WH provided the guidance as a co-supervisor. As a supervisor SP gratefully provided lab facilities and funding for the present work.

### Conflict of interest statement

The authors declare that the research was conducted in the absence of any commercial or financial relationships that could be construed as a potential conflict of interest. The reviewer, VM, and handling Editor declared their shared affiliation.
